# Hydrogels Based on Poly([2-(acryloxy)ethyl] Trimethylammonium Chloride) and Nanocellulose Applied to Remove Methyl Orange Dye from Water

**DOI:** 10.3390/polym13142265

**Published:** 2021-07-10

**Authors:** Karina Roa, Yesid Tapiero, Musthafa Ottakam Thotiyl, Julio Sánchez

**Affiliations:** 1 Departamento de Ciencias del Ambiente, Facultad de Química y Biología, Universidad de Santiago de Chile (USACH), Santiago 9160000, Chile; karina.roa@usach.cl (K.R.); yesidtm@gmail.com (Y.T.); 2 Department of Chemistry, IISER Pune, Dr. Homi Bhabha Road, Pune 411008, India; musmuhammed@gmail.com

**Keywords:** adsorption, fibrillated nanocellulose, hydrogel, methyl orange, polymer

## Abstract

Bio-based hydrogels that adsorb contaminant dyes, such as methyl orange (MO), were synthesized and characterized in this study. The synthesis of poly([2-(acryloyloxy)ethyl] trimethylammonium chloride) and poly(ClAETA) hydrogels containing cellulose nanofibrillated (CNF) was carried out by free-radical polymerization based on a factorial experimental design. The hydrogels were characterized by Fourier transformed infrared spectroscopy, scanning electron microscopy, and thermogravimetry. Adsorption studies of MO were performed, varying time, pH, CNF concentration, initial dye concentration and reuse cycles, determining that when the hydrogels were reinforced with CNF, the dye removal values reached approximately 96%, and that the material was stable when the maximum swelling capacity was attained. The maximum amount of MO retained per gram of hydrogel (q = mg MO g^−1^) was 1379.0 mg g^−1^ for the hydrogel containing 1% (w w^−1^) CNF. Furthermore, it was found that the absorption capacity of MO dye can be improved when the medium pH tends to be neutral (pH = 7.64). The obtained hydrogels can be applicable for the treatment of water containing anionic dyes.

## 1. Introduction

Pollutants released by industrial liquid waste affect the quality of water in water bodies. They can engender serious health effects in plants, animals, and humans. Wastewater pollutants include dyes, surfactants, oils, lubricants, organic solvents, petroleum derivatives, and pharmaceuticals such as antibiotics, anti-allergy, and hormones [1,2]. Artificial dyes, which are largely used in various industries such as textiles, food, cosmetics, leather, paper, and pharmaceuticals, are highly dangerous organic pollutants [3,4]. Dyes are mutagenic agents even at low concentrations and render an undesirable color to water bodies [5]. The presence of dyes in wastewater that drains into water bodies makes it difficult for light to penetrate natural water bodies and negatively impacts photosynthetic activity [6]. A representative artificial dye is methyl orange (MO, dimethylaminoazobenzenesulfonate), which is non-biodegradable in nature; besides, it is a water-soluble carcinogen, azo dye that is widely used in textile industries, printing paper manufacturing, textile laboratories, chemical research, pharmaceuticals and research laboratories [7]. It pollutes water at low concentrations; large volumes of MO are produced as waste.

The MO molecule has a bright orange color when dissolved in water, stable chemical structure due to the presence of azo (–N=N–) and aromatic groups (which are highly toxic, carcinogenic and teratogenic), and is harmful to the environment and organisms since it shows low biodegradability [8]. MO can lead to critical health issues, like cyanosis, vomiting, tachycardia, tissue necrosis, and jaundice, and has been declared carcinogenic and tumorigenic by the International Agency for Research on Cancer (IARC) and the National Institute for Occupational Safety and Health [9]. Additionally, it is an allergenic substance that can cause eczema upon contact with the skin. Its presence in living organisms is considered harmful and can lead to a significant increase in the activity of the azo-nitro-reductase enzymes, producing aromatic amines that may cause intestinal cancer [10,11].

It is difficult to eliminate and control the dye concentration efficiently through traditional treatment methods such as coagulation, sedimentation, chemical oxidation, and biological digestion [12]. In general, conventional water treatment methods generate large volumes of residual sludge, use excessive process times, and consume large amounts of energy [13]. Adsorption technology is considered one of the most effective methods because of its simplicity, low energy consumption, short treatment times, low generation of sludge, high efficiency, flexibility, and insensitivity to toxic substances.

Various materials have been used to adsorb MO, for example, activated carbon from natural sources [14], algae [15], hybrid materials with metal oxides [16], chitosan and hydrogen peroxide–treated anthracite sheets [17], and hypercrosslinked cyclodextrin networks in the form of nanofibrous membranes [18]. For example, Borsagli et al. designed and developed novel three-dimensional porous scaffolds made of *N*-acyl thiolated chitosan using 11-mercaptoundecanoic acid, with high adsorption capacities for the anionic MO dye in an aqueous medium [19]. Liu et al. prepared hydrogel particles of methacrylateethyltrimethylammonium chloride and acrylamide copolymer, with the ability to eliminate anionic dyes such as amaranth red, orange G, and MO reaching 94% efficiency [20]. Onder et al. prepared copolymer hydrogels of poly([2-(acryloyloxy)ethyl] trimethylammonium chloride-co-1-vinyl-2-pyrrolidinone) (p(AETAC-co-NVP)) which showed the ability to retain the MO and alizarin red S dyes through electrostatic interactions when the test pH values were 7.0 and 5.0, respectively [21], and Dalalibera et al. prepared hydrogels based on polyacrylic acid with the ability to absorb and selectively separate cationic and anionic dyes at a pH of 8.0 to 10.0 [22].

Recently, nanocomposites based on cationic polymers and nanocellulose have been prepared for application in water treatment. These materials have shown remarkable capabilities to remove oxyanions such as chromates and have improved mechanical properties [23], for example, Szekely et al. prepared nanocomposite hydrogels based on cellulose acetate, modified with the addition of small amounts of polymers of intrinsic microporosity and graphene oxide (GO), which demonstrated the ability to absorb neonicotinoid insecticidal pollutants in an aqueous medium [24]. Khan et al. prepared nanocomposite hydrogels with a porous 3D network structure based on cellulose-aluminum oxide nanoparticles-graphene oxide (GO) (Al_2_O_3_/GO), with application in the removal of fluoride ions from drinking water [25]. Hameed et al. prepared carboxymethyl cellulose/potato starch/amylum starch hydrogels where aluminum sulfate octahydrate was used as a cross-linking agent, which reached high capacity in the retention of heavy metals (cadmium, lead, and iron) from municipal drinking water [26].

In addition, nanocellulose has been used for the synthesis of hydrogels with applications in biomedicine, as in the case of the research work by Chen et al. They prepared fluorescent compound hydrogels of nitrogen-doped carbon points/cellulose nanofibrils (NCD/CNF-gel), where the mechanical properties were highlighted [27].

Several synthetic resins have been studied (such as Amberlite [28]), and the challenge now is to manufacture biomass-derived materials for removal of anionic dyes such as MO. There are not many papers on biomass-derived hydrogels that remove this dye, and that is why in this study hydrogels with ammonium groups reinforced with CNF were developed.

The aim of this study was to synthesize poly([2-(acryloyloxy)ethyl] trimethylammonium chloride) and poly(ClAETA) hydrogels containing fibrillated nanocellulose (CNF). The amount of crosslinkers, CNF, and initiators was optimized by studying the effects on the percentage yield of synthesis, degree of crosslinking, and water adsorption. Developing applicable hydrogels for adsorption and treatment of aqueous MO-containing wastes.

## 2. Materials and Methods

### 2.1. Materials

CNF was obtained by oxidation with 2,2,6,6-tetramethylpiperidine-1-oxyl (TEMPO) [29]. The synthesis was performed using a cellulose sample from Norweigan spruce wood, according to the procedure described by Dax et al. [30]. The suspension used in this study had a concentration of 10.79 mg g^−1^. Through conductometric titration analysis, it was determined that the CNF contained 0.96 mmol COO^−^ per gram of fiber.

[2-(acryloyloxy)ethyl]trimethylammonium chloride (ClAETA) solution (80 wt% in water; Aldrich, St. Louis, MO, USA), *N*,*N*-methylene-bis-acrylamide (MBA) (99%; Aldrich, St. Louis, MO, USA), ammonium persulfate (APS) (98%; Aldrich, St. Louis, MO, USA), NaOH (Merck, St. Louis, MO, USA), HNO_3_ (70%, Merck, St. Louis, MO, USA), HCl (36 v%; Merck, St. Louis, MO, USA), MO (85 vol% dye content; Sigma-Aldrich, St. Louis, MO, USA), and demineralized diethyl ether (99%, Merck, Milwaukee, WI, USA) were purchased and used in this study.

### 2.2. Synthesis of Hydrogels

The synthesis of the hydrogels containing CNF was performed via free-radical polymerization. For all experiments, the mass of ClAETA monomer was fixed at 5.0 g in 30 mL of deionized water type I. The synthesis was performed in a polymerization tube (Schlenk) under an inert atmosphere of nitrogen gas, which was immersed in a glycerine bath at 70 °C for 2.5 h. At the end of the reaction, the samples were dried in an oven (BIOBASE, model BOV-T50F, Jinan, Shandong, China) to obtain a constant weight. Subsequently, the samples were lyophilized (LABCONCO FREEZONE, Kansas City, USA).

**Figure 1:**
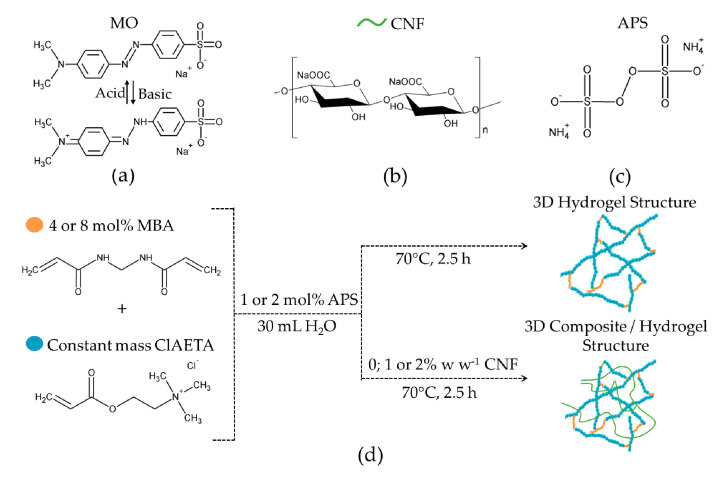
(**a**) Chemical structure of MO dye (molecular formula: C_14_H_14_N_3_O_3_SNa), (**b**) chemical structure of CNF, (**c**) chemical structure of poly(ClAETA), and (**d**) illustrative representation of the synthesis process of the exchange hydrogels obtained from this work.

Figure 1 shows the chemical structures of MO, poly(ClAETA), and the synthesis scheme.

### 2.3. Experimental Design

The synthesized materials were evaluated by analysis of variance (ANOVA) using Minitab 19 Statistical Software. The parameters studied in this experimental design were as follows: free-radical initiator (APS) (mol%), cross-linking agent (MBA) (mol%), and amount of CNF (wt%). Each parameter was considered with respect to the amount of ClAETA monomer. For this study, two test levels were used (minimum and maximum), as shown in Table 1. For the preparation, all possible combinations of the three factors were analyzed.

The combinations of the factors provide a design matrix of the type 2 × 2 × 3→12, thus assigning 12 treatments to be evaluated, as shown in Table 2. This method of experimental design is known as “Full Factorial Design (FFD)” [31]. An experiment is defined in which all possible combinations of factor configurations are tested and all possible interactions are determined. Full factorial designs are large compared to screening designs. Generally, a FFD is used when you have a small number of factors and levels, and you search for information on all possible interactions. In a FFD, an experimental run is performed for each combination of factor levels. The sample size is the product of the number of factor levels. FFDs are the most conservative of all design types.

The factorial experimental design for three factors (A, B, and C) facilitated the investigation of the individual and combined effects of A, B, C, AB, AC, BC, and ABC, based on two levels for each factor. Hence, seven effects were analyzed using ANOVA. In this test, it was assumed that the data followed a trend represented by the F statistic (Ronald Fisher). To ensure that the uncontrolled factors did not affect the results, the experiments were performed randomly. In addition, we analyzed whether the values found (yield of the reaction, cross-linking degree, and water absorption capacity) generated statistically significant effects, through standardized Pareto charts using the same software.

### 2.4. Theory Section: Determination of Yield of the Reaction, Cross-Linking Degree, and Water Absorption Capacity

For each of the synthesized materials, the reaction performance, degree of cross-linking, and water absorption were calculated according to the following procedure.

#### 2.4.1. Determination of the Reaction Yield

The yield of the polymerization reaction was determined by weighing the resulting freeze-dried sample (xerogel) and comparing it with the total mass of the reagents using Equation (1).

(1) $$\%Y=\frac{mass_{xerogel}\left(g\right)}{mass_{total}\left(g\right)}\times 100\%$$

where *mass_xerogel_* (g) is the mass of the xerogel in grams, and *mass_total_* (g) is the total mass of the reactive compounds.

#### 2.4.2. Determination of Water Absorption Capacity

To determine the maximum water absorption capacity of each of the synthesized hydrogels, 100 mg of xerogel was added to 80 mL of distilled water. The hydrated hydrogel was weighed and deposited in water. The water absorption capacity (%*WA*) was determined using Equation (2).

(2) $$\%WA=\left(\frac{W_{1}-W_{0}}{W_{0}}\right)\times 100\%$$

where *W*_1_ and *W*_0_ are the weights in grams of the swollen and initial hydrogels, respectively.

#### 2.4.3. Determination of the Effective Cross-Link Density of a Cross-Linked Structure

To determine the degree of cross-linking, 0.01 g of xerogel was taken in a petri dish, and 1 mL of demineralized ether type I was added. The swollen gels were dried superficially with filter paper and left to stand for 10 min, and the weight of the petri dish with the hydrated polymer material was recorded with XB220 Precisa Analytical Balance. The measurements continued until a constant weight was obtained for each sample. This weight was used to calculate the volume fraction *ν*_2m_ according to Equation (3):

(3) $$w=1-\frac{\%WA}{100\%}$$

where *w* is the weight fraction of polymer in swollen gel [32].

(4) $$C_{d}=\frac{mass_{xerogel,wet}\left(g\right)}{mass_{total,dry}\left(g\right)}$$

where *C_d_* is the equilibrium degree of swelling of the polymer in a gel sample that is swollen to equilibrium in water, *mass_xerogel,wet_* (g) is the wet mass of the xerogel in grams, and *mass_total,dry_* (g) is the total mass of the dry sample. The hydrated material was dried in an oven (BIOBASE model BOV-T50F) at 50 °C for 24 h. The dried mass of the material was measured again.

(5) $$\upsilon_{2m}=1/C_{d}$$

where *ν*_2*m*_ is the polymer volume fraction of the cross-linked polymer in the swollen gel.

(6) $$\frac{1}{\upsilon_{2m}}-1-\frac{\rho_{polymer}}{\rho_{water\;25\;{}^{\circ}C}}*\left(\frac{1}{w}-1\right)=0$$

where *ρ_polymer_* is the polymer density in g cm^−3^, and *ρ_water_* is the water density at 25 °C. By using the Excel solver function, it is possible to determine the value of the density of the polymeric part of the hydrogel using Equation (6).

(7) $$\bar{M_{C}}-\frac{\left(1-\frac{2}{\phi }\right)\times V_{1}\times \upsilon_{2m}^{\frac{2}{3}}\times \upsilon_{2r}^{\frac{1}{3}}}{\bar{\upsilon }\times \left(\ln\left(1-\upsilon_{2m}\right)+\upsilon_{2m}+\chi \times \upsilon_{2m}^{2}\right)}=0$$

where $\bar{M_{C}}$ is the average molecular weight of the network chains (g mol^−1^), *ν*_2*r*_ is the polymer volume fraction in the relaxed state, *V*_1_ (18,07 cm^3^ mol^−1^) 1 is the molar volume of the swelling agent (water, in this study), and $\bar{\upsilon }$ is the specific volume of the polymer. In this study, the reference value of cellulose is taken as 0.664 cm^3^ g^−1^) [33]. By using the Excel solver function, it is possible to determine the value of $\bar{M_{C}}$ using Equation (7).

(8) ϕ=3

where *ϕ* is functional at the cross-linking site [34,35].

(9) $$\chi =\frac{1}{2}+\frac{\upsilon_{2m}}{3}$$

where *χ* is the polymer–solvent interaction [35].

(10) $$\upsilon_{e}\left(\frac{mol}{cm^{3}}\right)=\rho_{polymer}/M_{C}$$

where $\upsilon_{e}$ is the effective cross-linking density of a cross-linked structure [36].

### 2.5. Physicochemical Characterization

#### 2.5.1. Fourier Transform Infrared (FTIR) Spectroscopy

Spectrum Two (UATR Two; Perkin Elmer) spectrophotometer with discs of KBr, in the spectral range of 4000–400 cm^−1^, was used for the FTIR measurements to determine the functional groups of the hydrogels.

#### 2.5.2. Scanning Electron Microscopy (SEM)

The surface morphology of the hydrogels was observed using scanning electron microscopy (SEM; Zeiss EVO MA 10 model, Oberkochen, Germany). The analyzed samples were previously hydrated with water until saturation.

#### 2.5.3. Thermogravimetric Analyses (TGA)

Thermobalance (STARe System Mettler Toledo, Greinfensee, Switzerland) equipment was used for the thermal gravimetric analysis, which was performed under a nitrogen gas atmosphere with a heating rate of 10 °C min^−1^ and a temperature range between 30 °C and 550 °C. A 250 mL min^−1^ flow rate of nitrogen gas was employed with aluminum as a reference material.

### 2.6. Adsorption Capacity of MO

The hydrogels were analyzed according to their ability to adsorb MO dye. An aqueous solution (water conductivity 3.15 μS/cm) of MO dye (150 mg L^−1^) was prepared, and 40 mL was used for each test. The hydrogel (50 mg) was placed in contact with 40 mL of aqueous MO dye, and the duration of the experiment was 300 min. The following hydrogels were selected for these tests: Hy01, Hy02, Hy03, Hy04, Hy07, Hy10, and Hy12.

First, the adsorption kinetics were determined using washed and unwashed hydrogel Hy12 after synthesis. Subsequently, the adsorption was studied as a function of time up to 300 min for Hy01 and Hy03; the adsorption capacity of the hydrogel was calculated every 15 min up to 60 min, then every 30 min up to 240 min, and finally at 300 min. Thereafter, the adsorption tests were performed simultaneously as mentioned above with Hy01, Hy02, and Hy03, by varying the amount of CNF in the hydrogel.

The adsorption tests were performed with Hy01 by varying the pH of the colored solution to acidic (pH 3.0) with HCl (0.1 mol L^−1^) and alkaline (pH 10.0) with NaOH (0.1 mol L^−1^). The MO dye concentration was measured using a UV-visible spectrometer (model BK-UV1800, BIOBASE) at a wavelength of 466 nm [37].

The amount of MO dye retained by the hydrogel was determined according to Equation (11):

(11) $$q=\frac{\left(C_{i}-C_{p}\right)\times V_{i}}{m_{x}}$$

where *q* is the adsorption capacity or amount in milligrams of MO retained per gram of sorbent (mg g^−1^); *C_p_* is the concentration of dye in the supernatant (mg L^−1^); *C_i_* is the initial concentration of dye in the supernatant (mg L^−1^); *V_i_* is the volume of the dye solution (mL); and *m_x_* is the mass of the xerogel (g).

The removal percentage %R of MO by the hydrogels was determined using Equation (12):

(12) $$\%R=\frac{C_{i}-C_{p}}{C_{i}}\times 100$$

where %*R* is the removal percentage; *C_p_* is the concentration of dye in the supernatant (mg L^−1^); and *C_i_* is the initial concentration of the dye in the supernatant (mg L^−1^).

The effect of the initial concentration of MO was studied by varying the concentrations between 50, 100, 200, 350, 500, 750, 1000, 1500, and 2000 mg L^−1^.

### 2.7. Adsorption Capacity of MO

To study adsorption and desorption cycles, adsorption was performed under the best conditions (pH, time, and concentration) and desorption was performed using 40 mL of HCl (0.1 mol L^−1^) as eluent during 30 min of constant agitation at 200 rpm. Each adsorption and desorption was then centrifuged for 10 min at 9000 rpm in a centrifuge, measuring the concentration of the supernatant by a UV-visible spectrometer (model BK-UV1800, BIOBASE, Jinan, Shandong, China).

### 2.8. Kinetic Model

To understand the dynamics of adsorption as a function of time, kinetic models described in the literature were used [38]. Equations (13) and (14) show the linear expression for pseudo-first order and pseudo-second order respectively.

(13) $$\ln\left(q_{e}-q_{t}\right)=\ln\left(q_{e}\right)-k_{1}t$$

(14) $$\frac{t}{q_{t}}=\frac{1}{k_{2}q^{2}{}_{e}}+\frac{1}{q_{e}}t$$

where $q_{e}$ (mg g^−1^) it is the sorption in equilibrium, $q_{t}$ (mg g^−1^) is the sorption at any time t, $k_{1}$ and $k_{2}$ ($\min^{-1}$) are the sorption constants of the pseudo-first-order and pseudo-second-order model.

## 3. Results

### 3.1. Synthesis of Hydrogels Modifying the Amount of Cross-Linker, Initiator, and CNF

Upon freeze-drying, the hydrogels had a granular texture and were slightly light brown. The nanocomposites synthesized with CNF had a slightly translucent appearance and, when freeze-dried, had granular characteristics and were light brown. Table 3 lists the reaction yields, water absorption, and effective cross-link density of the cross-linked structure of the 12 synthesized hydrogels.

The responses to the variations in the factors in the experimental design are explained below.

#### 3.1.1. Yield of the Reaction

Table 3 lists the reaction yields, water absorption, and effective cross-link density of the cross-linked structure of the 12 synthesized hydrogels.

The reaction yields of only four synthesized materials exceeded 90%: Hy06 > Hy09 > Hy12 > Hy03. All four hydrogels were prepared without the addition of CNF. Likewise, it can be observed that when the CNF concentration increased in the reactive mixture, the efficiency of the reaction decreased from approximately 30%, when 1% w w^−1^ CNF was added to the mixture, to approximately 17%, when 2% w w^−1^ CNF was added to the mixture. This indicated that CNF had an inhibitory effect on the growth of polymers through free-radical reactions (see Table 3).

#### 3.1.2. Water Absorption Capacity

The water absorption capacity of these hydrogels was high, indicating that they could effectively facilitate the removal of contaminants. From the results obtained for the %WA capacity, the following decreasing order can be observed: Hy03 > Hy10 > Hy04 > Hy01 > Hy09 (see Table 3). The highest water absorption capacity was achieved for the hydrogel that did not contain CNF; however, the remaining hydrogels that attained the highest %WA values contained 1% w w^−1^ CNF in their structures.

#### 3.1.3. Effective Cross-Link Density of a Cross-Linked Structure

The analysis of the effective cross-link density of a cross-linked structure revealed that five hydrogels exceeded the value of 6.0 × 10^−4^ mol cm^−3^: Hy04 > Hy05 > Hy10 > Hy09 > Hy01 (see Table 3). Except Hy12 and Hy06 all hydrogels contained CNF. The ν_e_ values were higher when 2% w w^−1^ CNF was added to the mixture.

### 3.2. Statistical Analysis

The results of the ANOVA on the yield of the reaction response (see Table S1a) indicated that the factors (A, B, and C) individually did not meet the criteria that represented a linear model of the data for the response of interest. Meanwhile, the value of the *p* statistic (significance value of 0.05) was found to be 0.037, which was less than 0.05. This result confirmed the prior result obtained through the F statistic. Therefore, the performance of the polymerization reaction for hydrogel synthesis was independent of factors A, B, and C. Furthermore, when analyzing factor B (CNF w w^−1^ %), it was observed that the value of the F statistic was greater than that of F_critical_ and the *p* statistic (0.008 < 0.05), considering the results obtained from the double and triple interactions of the factors. The values of the F statistic were less than the F_critical_ values, and the values of the *p* statistic were less than 0.05.

The ANOVA results for the response of the water absorption capacity (see Table S1b), including all the factors (individually as well as in their binary and ternary combinations), generated an F statistic value that was less than the F_critical_ statistic value, and the *p* values were greater than the significance value of 0.05. All parameters significantly influenced the water absorption capacity.

The ANOVA results for the response of the effective cross-link density of a cross-linked structure can be observed in Table S1c, where the F statistic value was greater than the F_critical_ value; similarly, the value of the *p*-statistic (0.027) was less than the significance value (0.05). Considering the results of the individual factors and their double combinations when comparing the values of the F statistic with their F_critical_ values, F < F_critical_. Furthermore, the values of the *p* statistic for these factors and their double combinations generated values less than 0.05; therefore, they were significant and influenced the response of the effective cross-link density of a cross-linked structure.

To establish the relationship between the investigated factors and yield of the reaction, water absorption capacity, and the effective cross-link density of the cross-linked structure, the data were graphically displayed using standardized Pareto charts. The changes in the level of a factor that influenced the different system responses were represented as the effects of the factor. The standardized Pareto chart compares the absolute values and the significance of the effects.

**Figure 2:**
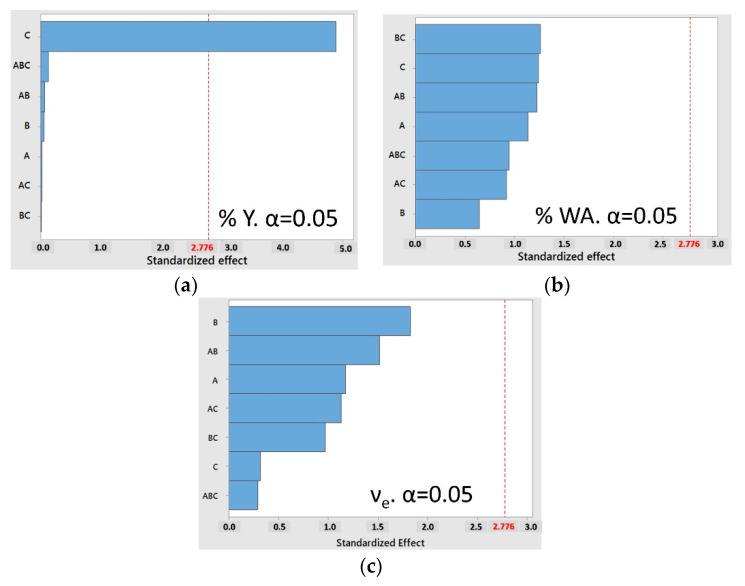
Result of the standardized Pareto analysis for the response variables (parameters A: MBA mol%; B: APS mol%; and C: CNF w w^−1^%): (**a**) yield of the reaction, (**b**) water absorption capacity, and (**c**) the effective cross-link density of the cross-linked structure.

Figure 2 shows standardized Pareto charts for the response variables, highlighting that the critical value obtained from the Student’s *t*-test statistic is *t*_0.25,8_ = 2.776.

From the ANOVA analysis for the three factors, it was found that, for the treatment of the data obtained for the reaction yield, only the CNF factor w w^−1^ % generated a value of the *p* statistic at 0.008 << 0.05; therefore, this factor significantly influences the performance results. Furthermore, the error had eight degrees of freedom, and we worked with α = 0.05; therefore, the critical value obtained from the Student’s *t*-test was *t*_0.25,8_ = 2.776. Figure 2a shows that the bar of the CNF factor w w^−1^% exceeds the critical value of 2.776; therefore, it was concluded that the reaction performance was affected significantly. Other factors did not significantly affect the yield of the reaction. The ANOVA analysis of the results obtained for the effective cross-link density of a cross-linked structure and the water absorption capacity also presented eight degrees of freedom in the error value; therefore, the critical value of the Student’s *t*-test statistic was *t*_0.25,8_ = 2.776. It can be observed from Figure 2b that none of the factors or the combination of these factors had a significant effect on the water absorption capacity, as none of the bars exceeded the critical value of *t*_0.25,8_ = 2.776.

The effective cross-link density of the cross-linked structure was not strongly affected by the interactions of the three factors (MBA mol% × APS mol% × CNF w w^−1^ %) because the value shown in Figure 2c does not exceed the critical value of *t*_0.25,8_ = 2.776.

The analysis of the effect of APS mol% in these tests revealed a random behavior. APS affects the length of the polymer chains, making them longer or shorter depending on the concentration used. Increasing the initiator percentage produces more free radicals that accelerate the chain termination reaction, resulting in shorter chains and thus increasing the possibilities for water entry [39]. The incorporation of CNF helps ensure that the hydration of the polymer does not lead to swelling drastically, since the fibrils become a part of the interpolymeric network when physically mixed and by electrostatic interactions, and continue to absorb significant percentages of water. An increase in hydrogel swelling is observed when the concentration of CNF is increased, which may be explained by the increased number of carboxyl groups in the hydrogels [40].

### 3.3. Physicochemical Characterization

#### 3.3.1. Fourier Transformed Infrared Spectroscopy

FTIR spectroscopy was performed to identify the functional groups of the polymers and the specific interactions between poly(ClAETA) and CNF, as well as to understand how this reinforcement interferes with the hydrogel base.

**Figure 3:**
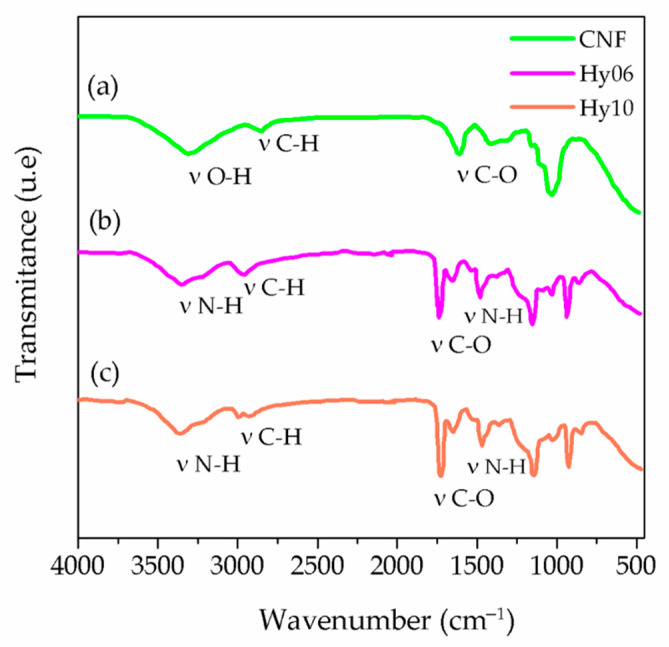
FTIR analysis of (**a**) CNF, (**b**) Hy06 (hydrogel without CNF), and (**c**) Hy10 (hydrogel with CNF).

Figure 3a shows the characteristic signal of CNF, where the characteristic band of the -OH stretching (3319 cm^−1^), band for -CH stretching (2900 cm^−1^), and the band with the signal of the -C=O (1610 cm^−1^) indicate the occurrence of -COOH groups in CNF, suggesting oxidation in the glucose ring of the hydroxyl group at the C-6 position [41,42]. Figure 3b,c shows the characteristic signals for two synthesized hydrogels, Hy06 and Hy10, corresponding to NH stretching (3357 cm^−1^) and disappearance of the vinyl group (C=C) (1200 cm^−1^), respectively. The carbon double bond signal disappeared, and the signals of the functional groups of the 2-(acryloyloxy) ethyl trimethylammonium chloride monomer (ClAETA) remained, corroborating the formation of the polymer [43,44]. The signals indicating the stretching of CH in the polymer (2977 cm^−1^), the carbonyl bond (C=O) (1736 cm^−1^ and 1726 cm^−1^), and the quaternary ammonium group (-N^+^ (CH_3_)_3_) (1500 cm^−1^) were also observed [44].

#### 3.3.2. Morphological Analysis by Scanning Electron Microscopy

**Figure 4:**
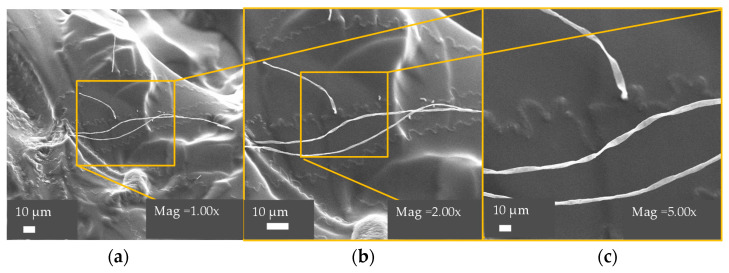
SEM images of Hy05 (2 wt% CNF) samples at (**a**) ×1000, (**b**) ×2000, and (**c**) ×5000 magnification. Scale bar: 10 µm.

The microstructural changes in the hydrogels were analyzed using SEM, and the micrographs are shown in Figure 4.

Figure 4 shows the structure of Hy05, which contained 2 wt% CNF. Separated fibers were observed on the sample surface; it is known that the surface of poly(ClAETA) (without CNF) has a smooth morphology, as reported in previous studies [43]. CNF was partially carboxylated, where the carboxylate groups interacted electrostatically with the quaternary ammonium groups present in the polymer, according to the SEM images. The CNF fibers were confirmed to be homogeneously distributed in the hydrogel network. Hy05 presented uniform roughness across the surface of the material; in addition, this physical mixture of CNF with the polymer chains helped in the stability and compactness of the structure [45]. In prior research studies, it has been reported that if one of the polymeric components has negatively charged functional groups, the formation of porosities is facilitated because of the repulsion forces between charges [40].

#### 3.3.3. Thermogravimetric Analysis

**Figure 5:**
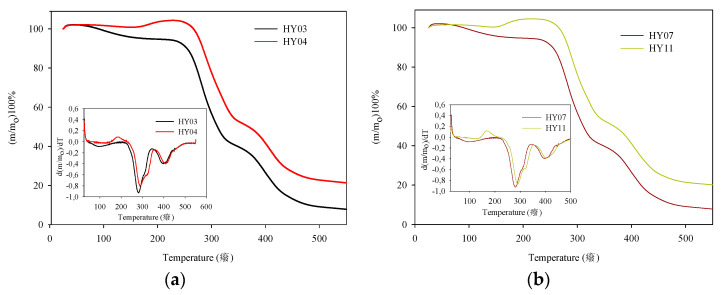
Thermogravimetric analysis TGA of the samples: (**a**) Hy03 and Hy04, (**b**) Hy07 and Hy11.

Thermogravimetric analysis is a technique that allows the evaluation of the thermal stability of synthesized poly(ClAETA) hydrogels and the determination of the effect generated by the addition of CNF in the hydrogels. Figure 5 shows the thermograms of hydrogels Hy03, Hy04, Hy07, and Hy11, which show a typical sigmoidal shape, indicating the weight loss in three stages. The first stage was recorded at temperatures around 100 °C, corresponding to the dehydration of water in the polymer and the elimination of humidity [46]. The second stage occurred at approximately 280–330 °C, corresponding to the thermal decomposition of the groups that protruded from the polymer chain. Similarly, a peak was observed at 390–410 °C, corresponding to exothermic reactions resulting from the decomposition of the ammonium salt [43].

Upon analysis of Hy03, which did not contain CNF, a decomposition was observed from 248.0 °C to 330.0 °C, resulting in a residual mass percentage of 45.1%; in the second stage, a residual mass percentage of 18.4% was obtained with respect to the initial mass; and finally, at 550 °C, only 8% of the residual mass of the hydrogel remained. In the case of Hy04, a rapid decomposition was observed that generated 58% of the residual mass in the second stage; in the third stage, a residual mass percentage of 26.7% was obtained; and finally, at 550 °C, the resulting value of the residual mass was 21.6%. It can be observed that there was a difference in stability between the hydrogels Hy03 and Hy04 at 18 °C, which could be due to the presence of CNF [47]. In the thermogram of Hy07, the first stage had a temperature range of 238–317.3 °C, which generated a residual mass percentage of 46.7%; in the third stage, 317.3 °C and 426 °C corresponded to a residual mass percentage value of 17.8%; and finally, at 550 °C, only 8% of the residual mass of the hydrogel remained.

Finally, the hydrogel Hy11 presented the second range of decomposition between the temperatures 256 °C and 330.2 °C, and the residual mass was 57.2%; between 330.2 °C and 434.3 °C, the residual mass was 24.8%. For this material, a thermal stability was achieved at approximately 8 °C. Analysis of Hy11 with Hy07 reveals that the main chain decomposition stage had a wider temperature range, which could be caused by the higher concentration of APS and CNF. Finally, at 550 °C, a residual mass percentage of 20.3% was obtained. The higher residual mass may be due to the higher amount of initiator, which can accelerate the cross-linking process during polymerization [48].

### 3.4. Adsorption Capacity of Methyl Orange Dye by Hydrogels

The functionality of hydrogel as a water dye adsorbent was evaluated. These tests were conducted using MO as the study molecule. For all the tests, the concentration was fixed at 150 mg L^−1^.

**Figure 6:**
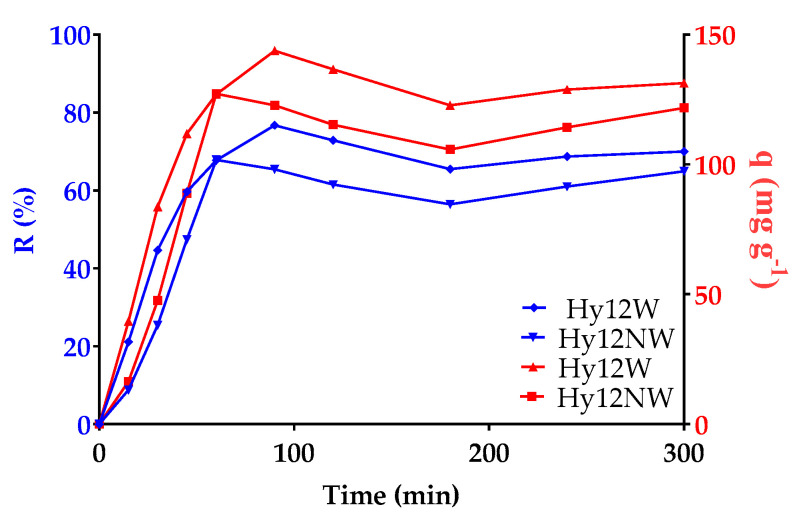
Dye retention and capacity of adsorption per gram of resin by hydrogel, as a function of time for Hy12NW and Hy12W at pH 7.64.

First, we studied the adsorption of dyes when the polymers were washed after synthesis, as well as the effects of this process on the result. For this test, we used Hy12. Figure 6 shows dye retention and capacity of adsorption per gram of resin for the washed (Hy12W) and unwashed (Hy12NW) hydrogel.

In the case of the Hy12W hydrogel, the minimum concentration of MO that remained in the solution at 90 min was 8.26%, compared to the case of Hy12NW, in which MO concentration was 28.13% at 60 min. Furthermore, as shown in Figure 6, Hy12W attained an optimal MO adsorption capacity of 96% at 90 min after the start of the experiment; however, the MO adsorption capacity was 37.55% at 300 min. In Hy12NW, an optimal MO adsorption capacity of 84.77% was attained at 60 min; at 300 min, the MO adsorption capacity was 81.16%. Thus, we concluded that despite washing, the hydrogels without CNF had a greater instability, which led to the desorption of the previously adsorbed dye.

**Figure 7:**
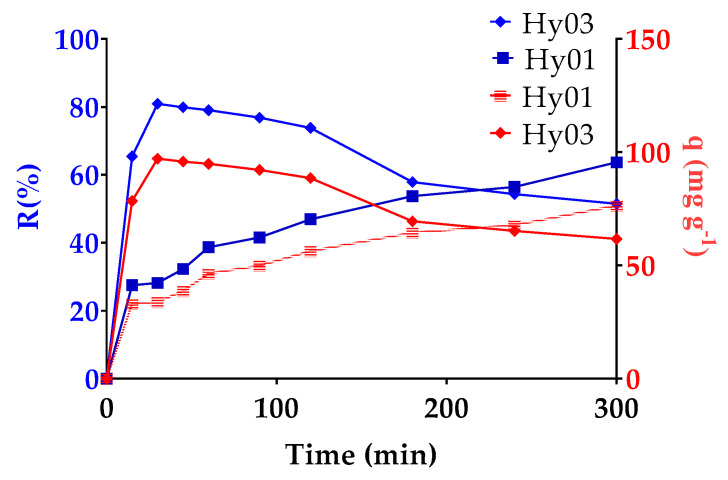
Dye retention and capacity of adsorption per gram of resin by hydrogel as a function of time for Hy01 and Hy03 at pH 7.64.

The removal of MO using two synthesized hydrogels, one with CNF and one without the reinforcement (Hy01 and Hy03, respectively), at a pH of 7.64 was evaluated with the same percentages of cross-linker and initiator. The results for the percentage of adsorption and the retention of the hydrogel are shown in Figure 7.

It was observed that for Hy03, the removal percentage at 30 min after being immersed in the MO solution was 80.92%, and adsorption decreased thereafter. Concurrently, it was observed that the concentration of dissolved dye in the solution increased, which could be due to the low stability of hydrogel. In general, it is observed that hydrogels absorb the contaminant until a plateau of stability or equilibrium is attained in the adsorption curve, which is maintained until the hydrogel reaches its maximum swelling capacity, whereupon the sample sorption starts decreasing [30]. In the case of the hydrogel with CNF, Hy01, a slower response to adsorption is observed, but this response is sustained when nearing 300 min with 76.41 mg of MO per gram of resin.

**Figure 8:**
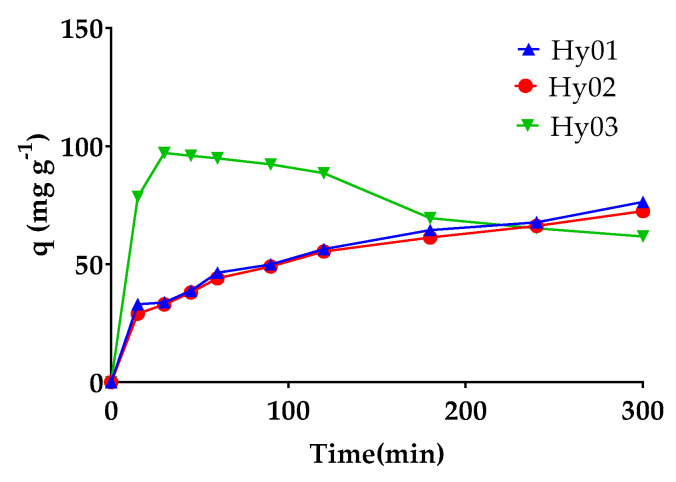
Removal of MO as a function of time when containing different concentrations of CNF at pH 7.64.

Comparing the amount of MO retained per gram of resin (q = mg MO g^−1^) and considering different percentages of CNF (see Figure 8), it can be ascertained that with CNF, the adsorption was slower but sustained with time, except in the case of Hy03 (control sample, without CNF), which retained a greater amount of MO in less time but lost the retained dye and returned it to the solution. In Hy01, containing 1% CNF, and Hy02, containing 2% CNF, we observed 76.41 mg of MO per gram of resin and 72.42 mg of MO per gram of resin after 300 min, respectively, which could be caused by the stability provided by the incorporation of CNF into the matrix.

#### 3.4.1. Effect of pH on MO Removal

**Figure 9:**
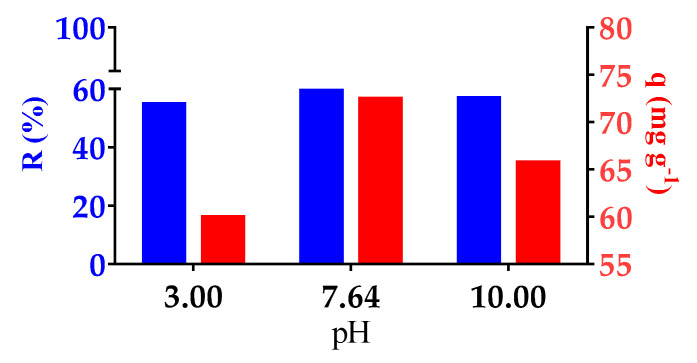
Removal of MO dye at pH 3, 7.64, and 10. *y*-axis: right—MO retention (%R); left—adsorption capacity (q).

The performance in terms of retention of the MO dye in the acidic and basic environment of Hy01 was analyzed. Figure 9 shows the results of the q response (concentration of MO dye absorbed in the hydrogel structure). There was a higher MO load at pH 7.64, with 76.41 mg of MO for each gram of resin; the MO load at pH 3 and 10 was 59.92 and 65.70 mg for each gram of resin, respectively. MO dye is a weak acid that is widely used as an indicator of pH change. It has a pKa value of 3.47; therefore, at pH 3.0, the sulfonate molecules in the dye are neutralized and the amino groups are protonated, generating a positive charge [28]. The positive charge of the quaternary ammonium group exerts electrostatic repulsion with the positive charge of the hydrogel. In contrast, in a basic medium, the molecules of MO have a completely ionized sulfonate group, and the amino component is uncharged; thus, the molecules in the dyes and the fixed quaternary ammonium groups in poly(ClAETA) can completely interact with each other electrostatically. In addition, CNF has carboxyl groups on its surface and a pKa of approximately 4.6. In an alkaline environment, the carboxylic acid groups in CNFs gradually change to carboxylic anions, leading to the interaction of weakened hydrogen bonds and increased electrostatic repulsion in the hydrogels [40].

#### 3.4.2. Adsorption as a Function of MO Concentration

**Figure 10:**
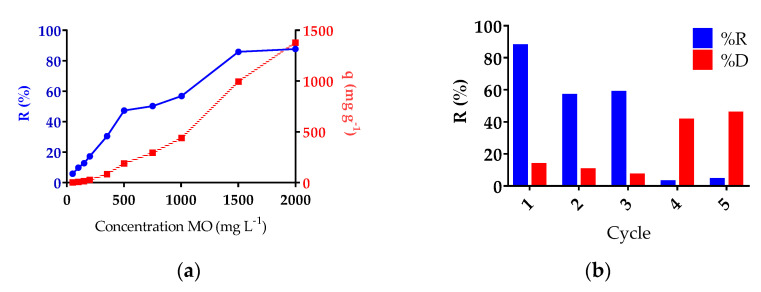
(**a**) Effect of MO concentration on the adsorption capacity of Hy01 at pH 7.64 with different concentrations of MO (mg L^−1^). (**b**) Effect of adsorption–desorption cycles on adsorption capacity (pH = 7.64; initial concentration: 2000 mg/L; and 50 mg adsorbent).

The initial concentration of the dye affects adsorption because it affects the mass transfer between the aqueous phase and the solid phase. For this reason studies were carried out varying the concentration between 50–2000 mg L^−1^ at pH 7.64 with an initial hydrogel mass of 50 mg (see Figure 10a). The adsorption capacity increased with increasing initial concentration; a similar situation was observed in previous investigations with this dye [49,50]. With respect to the retention percentage, it also increase as the concentration of MO increases. Equilibrium is achieved between 1500 and 2000 mg L^−1^, obtaining an maximum adsorption capacity of 1379 mg g^−1^ when the initial concentration is 2000 mg L^−1^. This high dye concentration was also studied by Onder et al., who using their hydrogel of [(2-(acryloyloxy)ethyl]trimethylammonium chloride-*co*-1-vinyl-2-pyrrolidone] hydrogel reached 905.6 mg g^−1^ [21]. Regenerability and reusability of the adsorbent are also very important, as they make the adsorption process economical. Figure 10b shows the adsorption–desorption cycles when hydrochloric acid was used as an eluent [51]. As the number of cycles increases, the adsorption capacity gradually decreases to 5% by the fifth cycle. In general, in our experimental conditions, the reuse is recommended up to the third cycle, since after this adsorption capacity decreases significantly. It is also noted that the hydrogel delivers low concentrations of MO up to the third desorption process.

#### 3.4.3. Kinetic Models

**Figure 11:**
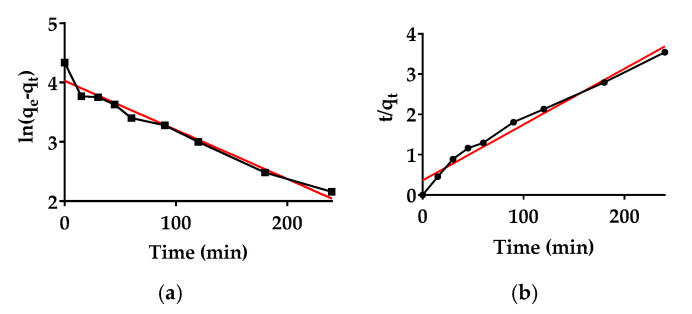
MO sorption kinetics: (**a**) pseudo-first-order and (**b**) pseudo-second-order kinetic model.

A study of adsorption kinetics is desirable because it provides information on the progress of adsorption and whether physical or chemical interactions predominate the process. The pseudo-first and pseudo-second-order kinetic parameter values for MO adsorption are presented in Table 4. The correlation coefficient criterion (highest value of R^2^) was used to describe the most suitable kinetic adsorption model [52]. According to the described criteria, MO adsorption for hydrogel Hy01 conforms to the pseudo-second-order kinetic model with an R^2^ value greater than 0.9733 (see Figure 11). Similar kinetic results were obtained in previously reported MO adsorption studies [7,19,53].

From a systematic study of the literature, it is clear that the textile industry is the main industry that generates large volumes of wastewater containing dyes, consuming about 100 L of water to process approximately 1 kg of textile material [54]. These are highly recalcitrant and biocompatible synthetic chemical compounds, considered as potential threats to human and environmental health [4]. About 3500 different types of synthetic dyes are used in the textile industry. The most commonly used dyes are anthraquinone and azo dyes and more than 60% of these dyes are reactive [55]. These chemical species are released through the industrial processes of dyeing and washing, among others [56], resulting in wastewater with high concentrations fluctuating between 350–1000 mg L^−1^ of dyes [4,57,58]. Most synthetic dyes are soluble in water, thanks to the ionizable groups that compose them, such as: -OH, -COOH, and -SO_3_H in acid dyes, and -NH_2_, -NHR, and -NR_2_ in basic dyes. It is estimated between 50% and 70% of the world production of 10,000 synthetic dyes (dyes and distinctive dyes used in the textile industry) corresponds to azo dyes, which represent the class of compounds most used in textile and food processes [10,11]. The pH value of the aqueous medium favors the ionization of the groups, depending on the pKa value of the chemical species in the solution. It is known that the average pH value of wastewater is 8.75 ± 1.29 [4], where the vast majority of dyes are in an ionic state. Regarding the applicability of hydrogels, they are materials that possess a number of functional groups suitable for dyes, and also offer the possibility of reuse/regeneration in sorption–desorption cycles by washing processes with acidic solutions for the anionic hydrogel and basic brine/NaCl for the cationic hydrogel. If it is not possible to regenerate the structures, these materials should be disposed of in the solid waste landfill, following the usual route for hazardous solid waste [59].

Table 5 shows a comparison of adsorption capacities of MO by biopolymer composites, highlighting the possibility of further developing this type of adsorbent materials with natural polymers, such as CNF, which was corroborated to improve the stability at the time of adsorption, giving the possibility of reusing the hydrogel in desorption–adsorption cycles.

Is important to advance in the development of bio-based materials to be tested in real applications in industry. The adsorption is inexpensive, simple, and easy to adapt. In addition, its treatment period is short, causes no pollution to the environment, and has been confirmed as one of the most promising technologies for removing dyes from wastewaters [50].

## 4. Conclusions

Nanocomposite hydrogels based on ClAETA were successfully synthesized by varying the concentrations of CNF, MBA, and APS. From the ANOVA analysis, it was observed that the concentration of APS significantly affects the performance of the hydrogel synthesis compared to the other factors. It was determined that the combination of the three factors significantly affected the degree of cross-linking because the APS affects the length of the polymeric chains formed, the MBA maintains the solidity and porosity of the hydrogel, and the APS provides stability and rigidity, and an increase in hydrogel swelling is observed when the concentration of CNF is increased, which may be explained by the increased number of carboxyl groups in the hydrogel. In contrast, all individual factors, in double or triple combination with each other, did not significantly affect the water absorption capacity.

In addition, in the microstructural analysis, the texture of the hydrogels was determined, and the CNF fibers were individually identified. The functional groups of the structures of the hydrogels can be determined by FTIR spectroscopic analysis. From TGA it was verified that the hydrogels containing CNF generated greater thermal stability compared to hydrogels with only poly(ClAETA). The surface morphology of the obtained hydrogels was observed by SEM and the incorporated CNF was observed.

In the application of the hydrogels to the absorption of the dye, it was observed that the hydrogels containing only poly(ClAETA) achieved removal values above 80% and then decreased, but these were unstable after reaching the maximum swelling capacity and tended to destabilize. In contrast, hydrogels with CNF, such as Hy01, had lower removal rates than those without CNF but were chemically and mechanically more stable, capturing 1379 mg of MO per gram of resin after 300 min. The reuse/regenerative hydrogel was tested and was found to be satisfactory in up to three cycles. Tests with pH variations indicated that the adsorption of MO was favored under neutral pH. Therefore, it can be concluded that the incorporation of CNF improves the MO adsorption as a function of time.

## Figures and Tables

**Table 1:** Factors to be studied and their respective levels, where −, +, and 0 indicate the minimum, maximum, and control levels respectively.

Levels			−	+	0
3 Factors	MBA (mol%)	A	4	8	
	APS (mol%)	B	1	2	
	CNF (% w w^−1^)	C	1	2	0

**Table 2:** Experimental design matrix (2 × 2 × 3→ 12) for the synthesis of ion exchange hydrogels. Treatment “c” indicates that the lowest level of testing has been completed.

Code	Treatment	Factors		
		A MBA (mol%)	B APS (mol%)	C CNF (% w w^−1^)
**Hy01**	1	4	1	1
**Hy02**	c	4	1	2
**Hy03**	0	4	1	0
**Hy04**	4	4	2	1
**Hy05**	5	4	2	2
**Hy06**	6	4	2	0
**Hy07**	7	8	1	1
**Hy08**	8	8	1	2
**Hy09**	9	8	1	0
**Hy10**	10	8	2	1
**Hy11**	11	8	2	2
**Hy12**	12	8	2	0

**Table 3:** Characterization results of the synthesized hydrogels: the reaction yields, water absorption, and effective cross-link density of the cross-linked structure of the 12 synthesized hydrogels.

Code	Treatment	%Y	%WA	$\upsilon_{e}\;(\mathrm{mol}\;{\mathrm{cm}}^{-3})$
**Hy01**	1	30.8	3119.0	7.28 × 10^−4^
**Hy02**	c	17.3	1818.0	1.47 × 10^−4^
**Hy03**	0	96.4	9567.7	5.23 × 10^−5^
**Hy04**	4	29.8	4631.0	2.73 × 10^−3^
**Hy05**	5	17.8	918.3	1.84 × 10^−3^
**Hy06**	6	101.4	826.0	4.79 × 10^−4^
**Hy07**	7	28.5	1318.4	5.58 × 10^−4^
**Hy08**	8	18.2	889.5	2.05 × 10^−5^
**Hy09**	9	101.1	2032.0	7.75 × 10^−4^
**Hy10**	10	30.6	5004.0	8.55 × 10^−4^
**Hy11**	11	19.0	882.2	4.06 × 10^−4^
**Hy12**	12	97.8	890.9	4.73 × 10^−4^

**Table 4:** The MO sorption data for the pseudo-first and -second-order kinetic model.

Hydrogel	K_1_ (min^−1^)	R^2^ Pseudo-First Order	K_2_ (g mg^−1^ min^−1^)	R^2^ Pseudo-Second Order
Hy01	0.0083	0.9595	0.0004111	0.9733

**Table 5:** Comparative table of maximum adsorption results.

Adsorbent	Qmax (mg g^−1^)	Ref.
Ppy^@^magnetic chitosan	95	[60]
three-dimensional (3D) porous scaffolds made of *N*-acyl thiolated chitosan using 11-mercaptoundecanoic acid	434.89	[19]
particles of methacrylateethyltrimethylammonium chloride (DMC) and acrylamide (AM) copolymer hydrogel	992.63	[20]
Chitosan/diatomite composite	35	[61]
Banana peel	21	[62]
Chitosan/organic rectorite-Fe_3_O_4_	5.6	[53]
Poly([2-(acryloyloxy)ethyl] trimethylammonium chloride), poly(ClAETA), hydrogels containing fibrillated nanocellulose (CNF).	1379.0	This study

## Data Availability

Exclude this statement.

## Supplementary Materials

The following are available online at https://www.mdpi.com/article/10.3390/polym13142265/s1, Table S1: ANOVA results, where the significance is 0.05: (a) yield of the reaction, (b) crosslinking degree, and (c) water absorption capacity. DF (degree of freedom of the data), SS (the sum of the squares of the data), MS (mean sum of the squares of the data), F (F-statistic), value-*p* (*p*-value) and F_critical_ (F-statistic critical value).
