# Supplementary material for: Hydrogels Based on Poly([2-(acryloxy)ethyl] Trimethylammonium Chloride) and Nanocellulose Applied to Remove Methyl Orange Dye from Water
Source: Polymers (Basel). 2021 Jul 10;13(14):2265. doi: 10.3390/polym13142265 (PMC8309228; doi:10.3390/polym13142265)
Supplement: Supplementary file 1 [file polymers-13-02265-s001.zip › polymers-1266548-supplementary.pdf]

polymers

MDPI

Supplementary Material: Hydrogels Based on Poly([2-(Acryloxy)Ethyl] Trimethylammo-nium Chloride) and Nanocellulose Applied to Remove Methyl Orange Dye from Water

Karina Roa [1], Yesid Tapiero [1], Musthafa Ottakam Thotiyl [2] and Julio Sánchez [1,*]

[1] Departamento de Ciencias del Ambiente, Facultad de Química y Biología, Universidad de Santiago de Chile (USACH), Santiago, 9160000, Chile; karina.roa@usach.cl (K.R.); yesidtm@gmail.com (Y.T.)
[2] Department of Chemistry, IISER Pune, Dr. Homi Bhabha Road, Pune 411008, India; musmuhammed@gmail.com
* Correspondence: julio.sanchez@usach.cl

Table S1. ANOVA results, where the significance is 0,05: a) Yield of the reaction, b) Crosslinking degree, and c) Water absorption capacity. DF (degree of freedom of the data), SS (the sum of the squares of the data), MS (mean sum of the squares of the data), F (F-statistic), value-p (p-value) and $F_{critical}$ (F-statistic critical value).

a) %Y

**Source**	**DF**	**SS**	**MS**	**F**	**value-p**	**$F_{critical}$**
Model	7	13167,5	1881,1	3,41	0,126	6,09
Linear fit	3	13156,1	4385,4	7,95	0,037	6,65
MBA mol%	1	0,2	0,2	0	0,984	7,71
APS mol%	1	1,4	1,4	0	0,962	7,71
FNC w/w%	1	13154,4	13154,4	23,85	0,008	7,71
Adjustment of 2-term interactions	3	2,1	0,7	0	1	6,59
MBA mol%*APS mol%	1	2	2	0	0,955	7,71
MBA mol%*FNC w/w%	1	0,1	0,1	0	0,989	7,71
APS mol%*FNC w/w%	1	0	0	0	0,995	7,71
Adjustment of 3-term interactions	1	9,2	9,2	0,02	0,903	7,71
MBA mol%*APS mol%*FNC w/w%	1	9,2	9,2	0,02	0,903	7,71
Error	4	2206,5	551,6			
Total	11	15374				

b) %WA

**Source**	**DF**	**SS**	**MS**	**F**	**value-p**	**$F_{critical}$**
Model	7	50879982	7268569	1,16	0,471	6,09
Linear fit	3	20411878	68033959	1,08	0,452	6,65
MBA mol%	1	8106756	8106756	1,29	0,32	7,71
APS mol%	1	2606106	2606106	0,41	0,555	7,71
FNC w/w%	1	9699016	9699016	1,54	0,282	7,71
Adjustment of 2-term interactions	3	24843153	8281051	1,32	0,385	6,59
MBA mol%*APS mol%	1	9481223	9481223	1,51	0,287	7,71
MBA mol%*FNC w/w%	1	5291310	5291310	0,84	0,411	7,71
APS mol%*FNC w/w%	1	10070620	10070620	1,6	0,274	7,71
Adjustment of 3-term interactions	1	5624950	5624950	0.89	0,398	7,71
MBA mol%*APS mol%*FNC w/w%	1	5624950	5624950	0,89	0,398	7,71
Error	4	25151738	6287935			
Total	11	76031720				

c) %Cd

**Source**	**DF**	**SS**	**MS**	**F**	**value-p**	**$F_{critical}$**
Model	7	0,000005	0,000001	1,34	0,154	6,09
Linear fit	3	0,000002	0,000001	1,6	0,775	6,65

MBA mol%	1	0,000001	0,000001	1,4	0,728	7,71
APS mol%	1	0,000002	0,000002	3,3	0,451	7,71
FNC w/w%	1	0	0	0,1	0,614	7,71
Adjustment of 2-term interactions	3	0,000002	0,000001	1,5	0,177	6,59
MBA mol%*APS mol%	1	0,000001	0,000001	2,3	0,209	7,71
MBA mol%*FNC w/w%	1	0,000001	0,000001	1,3	0,138	7,71
APS mol%*FNC w/w%	1	0	0	0,94	0,184	7,71
Adjustment of 3-term interactions	1	0	0	0,08	0,027	7,71
MBA mol%*APS mol%*FNC w/w%	1	0	0	0,08	0,027	7,71
Error	4	0,000002	0,000001			
Total	11	0,000007				
